# Cost analyses of obesity in Canada: scope, quality, and implications

**DOI:** 10.1186/1478-7547-11-3

**Published:** 2013-02-08

**Authors:** Bach Xuan Tran, Amrita V Nair, Stefan Kuhle, Arto Ohinmaa, Paul J Veugelers

**Affiliations:** 1 School of Public Health, University of Alberta, Edmonton, AB, Canada; 2 Department of Pediatrics, Obstetrics & Gynecology, Dalhousie University, Halifax, NS, Canada

**Keywords:** Obesity, Overweight, Economic, Cost, Intervention, Canada

## Abstract

**Background:**

Rapid changes in lifestyle have led to a global obesity epidemic. Understanding the economic burden associated with the obesity epidemic is essential to decision making of cost-effective interventions. This study reviewed costs of obesity and intervention programs in Canada, assessed the scope and quality of existing cost analyses, and identified implications for economic evaluations and public health decision makers.

**Methods:**

A systematic search of costs associated with obesity or intervention program in Canada between 1990 and 2011 yielded 10 English language articles eligible for review.

**Results:**

The majority of studies was prevalence-based or top-down costing; 40% had excellent quality assessed using the Quality of Health Economic Study scale. The aggregated annual costs of obesity in Canada ranged from 1.27 to 11.08 billion dollars. Direct costs accounted for 37.2% to 54.5% of total annual costs. Between 2.2% and 12.0% of Canada's total health expenditures were attributable to obesity. The average annual physician cost of overweight male ($ 427) and female ($ 578) adults was lower than that of obese male ($ 475) and female ($ 682) adults; this cost differential across weight status groups was comparable to that found in adolescents. The cost for implementation and maintenance of a school-based obesity prevention program was $ 23 per student.

**Conclusions:**

We observed high costs associated with overweight and obesity and modest costs for obesity prevention programs; however, no cost-effectiveness study of obesity interventions has been performed in Canada. Cost-effectiveness analyses of preventive programs that constitute incidence-based life-time modeling of costs and health outcomes from societal perspective are urgently needed.

## Review

### Background

Obesity has rapidly developed into a major global public health challenge. In Canada, 24% of adults are obese and the rates of childhood obesity nearly tripled over the last two decades [1]. Childhood obesity is associated with obstructive sleep apnea, mental health problems, asthma, otitis media, and cardiovascular risk factors [2,3]. Obesity frequently tracks from childhood into adulthood and increases the risk of developing chronic diseases, including type 2 diabetes mellitus, cardiovascular disease and some types of cancer [4]. The negative health consequences of obesity place a substantial economic burden on the health care system and society [5].

Economic evidence is indispensable to evaluate the burden of illness and inform health policy development [6]. Obesity is associated with poorer health status, more frequent use of health care services, and increased direct health care costs [7,8]. Moreover, losses of productivity and healthy life-years due to absenteeism, co-morbidities, disability, and premature mortality are substantial indirect costs placed on individuals, their families, and society [9]. Evaluating the cost of obesity is essential to facilitate prioritization and resource allocation decisions on obesity prevention programs [10]. In addition, economic evaluations are essential to identify cost-effective and cost-saving obesity interventions towards the sustainability of the public health systems at provincial and federal levels [11,12].

There has been a growing body of literature on the assessment of economic burden of obesity in various settings [10,12]. Recent reviews on the topic examined the economic consequences of childhood obesity on health care systems [13], obesity costs in different models of health care systems [5], direct costs of obesity [14], and the cost-effectiveness of obesity interventions [12]. However, differences in health care financing and the heterogeneity in costing approaches hamper comparisons across countries, and call for country-specific reviews. In Canada, specifically, the publicly funded, single-payer health care system facilitates comprehensive access to health care services. There have been several interventions proven effective in controlling childhood and adulthood obesity [15-17]. While scaling up these measures is necessary, decision makers are also interested in economic returns of allocating scare resources on competing health and social problems [6]. To date, little evidence is available about the costs, cost-effectiveness, and cost-savings of these programs in Canadian settings [6]. The present study is a part of a greater effort to develop a framework for economic evaluations of obesity interventions. We aimed to review costs of obesity and intervention programs in Canada, assessed the scope and quality of existing cost analyses, and identified implications for economic evaluations and public health decision makers.

### Methods

#### Literature search and study selection

We searched for journal papers, conference abstracts, and research reports in MEDLINE (via PubMed) and government or organization’s websites in the period from 01 January 1990 to 01 May 2012, regardless of their publication status. We also consulted with experts in the field to identify additional relevant studies or reports. The search terms used for PubMed searches are shown in Table 1. We retrieved the titles and abstracts of these publications for screening. Reference lists of included studies were searched for additional potentially eligible studies. We contacted the authors if there was any annex or supplemental analysis of the papers that were not presented.

**Table 1:** Search strategy

**Search terms**	**Aims**	**Results**
(((((cost[Title/Abstract]) OR costing[Title/Abstract]) OR expenditure [Title/Abstract]) OR economic[Title/Abstract]) OR financial[Title/Abstract]) AND obesity[Title/Abstract]) OR overweight[Title/Abstract]) AND Canada[Title/Abstract]	Costs of obesity	Initial: 295 Selected: 9
	Cost of prevention program	Selected: 1

#### Inclusion criteria and selection of studies

All studies that performed any type of cost analysis (including but not limited to cost-of-illness, costs of health care services or prevention programs) related to excess weight in adults or children in Canada were eligible for inclusion in this review.

Two researchers (B.T. and A.O.) independently reviewed the retrieved titles and abstracts. For potentially relevant articles the full-text was obtained and reviewed by both reviewers for possible inclusion in the study. Disagreement between reviewers was solved by discussion. No third party adjudication was necessary.

#### Data extraction and quality appraisal

Full texts of all selected studies were retrieved and data were extracted using a standardized data extraction form. The form included study details (authors, year, and objectives regarding costs), costing approach (scope, data source, perspective, assumption, and year of cost determination), results (obesity measures, and cost estimates), strengths and limitations, and the quality scoring. For publications that reported similar results of the same work, we selected the most comprehensive paper or report to avoid duplications in the database.

Two researchers (B.T. and A.O.) assessed the quality of selected studies using the Quality of Health Economic Studies (QHES) scale [18]. The QHES consists of 16 criteria in the form of Yes/No questions that were selected by health economics experts. Each question has a weighted point value that creates a band score between 0 and 100. The QHES has been validated and shown to be convergent to other instruments such as the British Medical Journal checklist and the Consensus Health Economic Criteria list [19,20]. Compared to traditional non-quantitative classifications of studies’ quality, QHES is preferable given its summary score constructed by weighted criteria [18]. The score enables reviewers to directly compare and rank studies according to their quality.

#### Data analysis

Data are presented as total costs in Canadian Dollar (CDN$) (unless stated otherwise) and stratified by age and sex where available. Quality of studies was rated independently by two reviewers (B.T. and A.O.). Disagreement between reviewers was solved by discussion. No third party adjudication was necessary. Since no threshold for interpreting the QHES exists, we arbitrarily set a score of 90 and above as excellent quality, and a score of 75 to 90 as good quality.

### Results

The literature search was performed in May 2012 (search terms: cost, costing, expenditure, economic, financial, obesity, overweight, and Canada). The search yielded 295 articles from PubMed and 15 research reports from other internet sources. Applying the inclusion criteria, 10 studies were selected, including nine cost-of-illness (COI) analyses and one cost analysis of an obesity prevention program.

### Scope of cost analyses

Of 10 cost analyses, there were five federal and five provincial estimates (Ontario: n=3; Nova Scotia: n=2). The majority of selected studies examined costs for health care in adults (n=8), while few evaluated the health care costs for adolescents (n=1) and children (n=1). There was only one study that evaluated the costs of an obesity prevention program in children [21].

Based on the data used, two types of COI analyses can be differentiated: prevalence-based analyses and incidence-based analyses. Prevalence-based COI studies determine the direct cost and production losses attributable to all cases in a given year while incidence-based COI studies estimate the present value of the lifetime costs of an illness from onset to conclusion for cases first diagnosed within the study year. The majority of selected studies (n=8) were prevalence-based COI studies; only one study was an incidence-based COI analysis [7].

All studies used a top-down approach which multiplied health care unit costs by size of population. Five studies estimated costs at the population level, while the four other studies analyzed data of individuals. The scope of all five population estimates included direct health care costs, and four of them, except Bimingham et al., also estimated indirect costs, such as losses in productivity from a societal perspective (Table 2). Of the four individual analyses, the scope was more focused on direct medical service costs and health care utilization of overweight and obese individuals from the perspective of a provincial health care system (n=2) or the third party payer (n=2). The studies determined physician costs (n=4), and health services costs (e.g. costs of hospital stays or laboratory tests) (n=2). Details of cost components included in these analyses are shown in Table 2.

**Table 2:** Profile of selected studies

**Author, year**	**(Finkelstein 2001) **[24]	**(Patra, Popova et al. 2007) **[23]	**(Birmingham, Muller et al. 1999) **[22]	**(Tarride, Haq et al. 2012) **[27]	**(Janssen, Lam et al. 2009 2**[8]	**(Katzmarzyk and Janssen 2004) **[28]	**(Moffatt, Shack et al. 2011) **[26]	**(Anis, Zhang et al. 2009) **[11]	**(Kuhle, Kirk et al. 2011) **[7]	**(Ohinmaa, Langille et al. 2011)**[21]
**QHES score**	82	77	86	94	85	85	85	97	94	91
**Year of cost determination**	1994	1997	1997	2000	2000	2001	2005	2006	2006	2009
**Settings**	Ontario	Canada	Canada	Ontario	Ontario	Canada	Canada	Canada	Nova Scotia	Nova Scotia
**Objective regarding costs**	To compute direct estimates of the costs of physicians' services in Ontario in relation to Body Mass Index (BMI) and smoking	To estimate the economic cost of chronic disease in Canada	To estimate the direct costs related to the treatment of and research into Obesity in Canada in 1997.	To present an overview of the human and economic burden associated with BMI categories in Ontario, Canada, costs associated with hospitalization, same day procedures and physician visits	To perform an obesity cost-of-illness analysis for individuals living in the province of Ontario, Canada	To estimate the direct and indirect economic costs of physical inactivity and obesity in Canada in 2001	To estimate the direct and indirect costs associated with overweight and obesity	To estimate the economic burden of illness because of overweight and obesity in Canada	To assess health service use and costs across categories of weight status	To estimate the costs associated with implementing and maintaining comprehensive school health.
**Approach**	Prevalence-based	Prevalence-based	Prevalence-based	Prevalence-based	Prevalence-based	Prevalence-based	Prevalence-based	Prevalence-based	Incidence-based	Top-down costing
**Data source**	NPHS 1995/6.	Literature searches	- NPHS 1994/5.	- CCHS 1.1.	- CCHS 2000-1.	- CCHS 2001.	- CCHS 2004/5.	- National Health Expenditure Database.	2003 Children’s Lifestyle and School Performance Study (CLASS).	Accounting information of all schools in the Annapolis Valley Health Promoting Schools (AVHPS)
	Ontario Health Insurance Plan.		- EBIC 1993.	- Ontario Health Insurance Program (OHIP).	- Ontario Health Insurance Plan (OHIP).	- EBIC 1993, 1998.	- NPHS, CCHS, Literature searches (RRs).	- EBIC.	Medical Services Insurance database	
			- Health expenditure from Health Canada.	- Discharge Abstract Database, Inpatient and Day Procedure.			- EBIC 2000.		.CIHI Discharge Abstract Database.	
									Nova Scotia Atlee Perinatal Database.	
**Number of comorbidities**		8	10			8	22	18		
**Sample size**	2,170			28,797	27,478				4,380	
**Perspective**	Third-party payer	Societal	Societal	provincial health system	third-party payer	Societal	Societal	Societal	Provincial health care system.	Program manager
**Direct Costs**	Physician Costs	hospital care, specialized treatment, physician care, prescription drugs, and additional direct health expenditures	Hospital care, physician services, services of other health professionals, drugs, other health care and health research.	DAD-IP: costs of inpatient hospital stays.	Physician	hospital care expenditures, drug expenditures, physician care expenditures, costs for care in other institutions, and additional direct health expenditures	Hospital care, drugs, physician care, institutional care, and additional direct costs such as capital investments, public health, and research.	Hospital care, physician services, services provided by other health professionals, drugs, health research and other health care	MSI: physician (incl. emergency room visits) Aggregate costs of health care episodes for physician (birth-2006) visits and hospitalizations (2003-2006)	
				DAD-DP: costs of day procedures.						
				OHIP: physicians and nonhospital Laboratories costs.						
**Indirect costs**		Mortality costs, morbidity costs due to long and short-term disability.				Mortality costs, morbidity costs due to long and short-term disability	Morbidity costs due to long and short-term disability.	Morbidity costs due to long and short-term disability.		
**Strengths/ Limitations in Costs Estimates**	Insufficient direct costs and indirect costs.	Not available.	Indirect cost excluded.	Drug costs, costs associated with other non-physician healthcare providers or indirect costs were not included..	Insufficient direct costs (only physician cost). Indirect costs were not included.	Both direct and indirect costs included.	Excluded costs:	Meta-analysis of relative risks of chronic conditions.	Lack of drug prescription costs.	Donations, volunteers contribution were not fully recorded and costed.
	Direct measures at individual level.			Direct measures at individual level		Meta-analysis of relative risks of chronic conditions	Out-of-pocket costs not reimbursed, morbidity costs.		Direct measures at individual level.	
	Self-reported BMI		Self-reported BMI.	Self-reported BMI.	Self-reported BMI	Measured BMI.	Measured BMI.	Measured BMI.	Measured BMI.	
				Overweight (BMI>=25) – 35 %	Adults – Overweight – 35. 85%	Obese (BMI>=30) - 14.7%	Overweight (BMI>=25) - 35.7%	Overweight (BMI>=25)	Overweight (BMI>=25) – 23%	
**Obesity measures and prevalence**	Overweight and Obese - 58.6%	Not stated.	Obese (BMI>=27) – 13.5%	Obese (BMI>=30) - 17%	Obese – 16.95%Adolescents - Overweight –		Obese (BMI>=30) - 25.2%;	Obese (BMI>=30).	Obese (BMI>=30) - 10%	
					15%Obese – 5.3%					
**Results**	$65 M	The total direct cost of obesity in Canada in 1997 was estimated to be between $2.1 billion to $11 billion (or between $64.4 and $343.4 per capita) ~ 2.4% to 12% of the total health care expenditures.	Total direct cost: $1.8 B (0.8-3.5 B) ~2.4% of the total direct health care expenditures in Canada in 1997.	One year total physician, hospitalization, day procedure costs: Normal: $708.0 ($668.2, $752.4)	*Adults:* Overweight: Male: 427 (397, 457) $/y, Female: 578 (542, 613) $/y ; Obese: Male: 475 (434, 518) $/y, Female: 682 (639, 736) $/y.	Physical inactivity (2.6% total health care costs in Canada): Total: $5.3 B; Direct: $1.6 B; Indirect: $3.7 B	Total $1.27 B.	Direct Costs - $ 6.0 B (65.7% attributable to Obesity) ~ 4.1% of the total direct health expenditures in Canada in 2006.	Population: 295 (133; 629)**;** Normal weight: 275 (128; 598)**;** Overweight: 298 (136; 600)**;** Obese: 356 (140; 721).	The annual public funding to AVHPS to implement and maintain CSH totaled $344,514, which translates, on average, to $7,830 per school and $22.67 per student
	The mean per capita cost of physicians' services in Ontario increased by $8.90 (95% CI: $1.90-$15.60) for each unit increase in BMI			Underweight: $746.0 ($652.0,	*Adolescents:* Comparable for normal-weight and overweight/ obese ($233/ y)	Obesity (2.2% total health care costs in Canada): Total: $4.3 B; Direct: $1.6 B; Indirect: $2.7 B.	Direct cost: $630.1M	Indirect Costs - $5.0 B ~4.2% total health expenditure in Canada in 2006.	Lifetime physician cost: Population: 2201 (1 449; 3 370) **;** Normal weight: 2147 (1 428; 3 297) **;** Overweight: 2309 (1 463; 3 315) **;** Obese: 2504 (1 694; 3 725)	
							Indirect cost: $643.8M			
				Overweight: $690.3 ($648.2, $736.4)						
				Obesity: $884.1 ($806.1, $953.8)						

Six of the studies described the thresholds for classifying weight status: five used BMI cut-offs of ≥25 kg/m2 and ≥30 kg/m2 for overweight and obesity, respectively, while one study used an obesity threshold of BMI ≥27 kg/m2 [22].

#### Quality of the evidence

The QHES scores ranged from 77 to 97 (Table 3). The proportion of studies of good or excellent quality was 40% and 60%, respectively. A higher average score was seen in health services cost analyses (mean ± SD = 88.8 ± 6.2) compared to COI studies (86.0 ± 7.1). Table 4 illustrates a breakdown of QHES responses by question. The majority of studies met quality criteria defined by the QHES; 13/16 criteria were positively rated in more than 90% studies. The low positive response rate was seen in the following questions: Question 5 (50%), “Was uncertainty handled by (1) statistical analysis to address random events, (2) sensitivity analysis to cover a range of assumptions?”; Question 6 (10%), “Was incremental analysis performed between alternatives for resources and costs?”; Question 16 (70%), “Was there a statement disclosing the source of funding for the study?”. The corresponding scores of question 5, 6, and 16 in the total QHES score was 9, 6, and 3, respectively.

**Table 3:** QHES score by types of studies

**Type of studies**	**n**	**QHES score**				**Classification**	
		**Mean**	**SD**	**Min**	**Max**	**Excellent**	**Good**
All studies	10	87.1	6.1	77	97	40%	60%
Macro estimates	5	86.0	7.1	77	97	20%	80%
Micro estimates	4	88.8	6.2	82	94	50%	50%
Cost of intervention	1	91					

**Table 4:** Percentage of responses by QHES question

	**QHES**	**% Reponses**
1	Was the study objective presented in a clear, specific, and measurable manner?	100%
2	Were the perspective of the analysis (societal, third-party payer, etc.) and reasons for its selection stated?	100%
3	Were variable estimates used in the analysis from the best available source (i.e., randomized control trial - best, expert opinion - worst)?	100%
4	If estimates came from a subgroup analysis, were the groups prespecified at the beginning of the study?	100%
5	Was uncertainty handled by (1) statistical analysis to address random events, (2) sensitivity analysis to cover a range of assumptions?	50%
6	Was incremental analysis performed between alternatives for resources and costs?	10%
7	Was the methodology for data abstraction (including the value of health states and other benefits) stated?	100%
8	Did the analytic horizon allow time for all relevant and important outcomes? Were benefits and costs that went beyond 1 year discounted (3% to 5%) and justification given for the discount rate?	100%
9	Was the measurement of costs appropriate and the methodology for the estimation of quantities and unit costs clearly described?	90%
10	Were the primary outcome measure(s) for the economic evaluation clearly stated and did they include the major short-term was justification given for the measures/scales used?	100%
11	Were the health outcomes measures/scales valid and reliable? If previously tested valid and reliable measures were not available, was justification given for the measures/scales used?	100%
12	Were the economic model (including structure), study methods and analysis, and the components of the numerator and denominator displayed in a clear, transparent manner?	90%
13	Were the choice of economic model, main assumptions, and limitations of the study stated and justified?	100%
14	Did the author(s) explicitly discuss direction and magnitude of potential biases?	100%
15	Were the conclusions/recommendations of the study justified and based on the study results?	100%
16	Was there a statement disclosing the source of funding for the study?	70%

#### Costs analyses of obesity and obesity prevention programs

Table 5 summarizes the economic burden of overweight and obesity in the Canadian settings. The aggregated annual costs of obesity in Canada ranged from 1.27 to 11.08 billion dollars. In those studies that presented both direct and indirect costs, the total direct costs accounted for 37.2% to 54.5% of total annual costs. In most studies, direct and indirect costs accounted for approximately 2 to 4% of the total health care expenditure. Noticeably, Patra et al. estimated the total costs of obesity ranged from 2.4% to 12% of total health care expenditure in Canada in 1997 [23].

**Table 5:** Summary of economic burden of excess weight in Canadian settings

**Settings, Year**	**# of comorbidities**	**Direct cost**	**% Direct/ total cost**	**Indirect cost**	**Total**	**% total Canada’s direct health care expenditure**
Canada, 1997	10	1.800			1.800	
Alberta, 2005	22	0.630	49.5%	0.644	1.274	
Canada, 2001	7	1.600	37.2%	2.700	4.300	2.2
Canada, 2006	18	6.000	54.5%	5.000	11.000	4.1
Ontario, 2000		0.175			0.175	
Ontario, 1994		0.650			0.650	
Ontario, 2000					N/A	
Nova Scotia, 2006		0.295			0.295	
Canada, 1997		11.082			11.082	12

In health service costs analyses, the average annual physician cost of overweight male ($ 427) and female ($ 578) adults was lower than that of obese male ($ 475) and female ($ 682) adults in 2000; this cost differential across weight status groups was comparable to that found in adolescents [8]. Costs for physician services were estimated to increase by $ 9 for each unit increase in BMI in 1994 [24]. Tarride et al. estimated the physician, hospitalization and day procedures costs of normal weight, underweight, overweight, and obese adults in 2000 to be $ 708, $ 746, $ 690 and $ 884. Kuhle et al. reported the 2006 physician costs of normal weight, overweight, and obese children to be $ 275, $ 298, and $ 356, respectively [7].

In the only cost analysis of a school-based obesity prevention program in Canada, Ohinmaa et al estimated the costs for the school board-wide implementation and maintenance of the program at $ 344,514, or $ 7,830 per school and $ 23 per student [21].

#### Relative Risks (RR) and Population Attributable Fractions (PARF) of Obesity-related diseases

Estimates of relative risks and population attributable fractions of health conditions associated with overweight and obesity are key components of COI studies. The number of obesity-related health conditions ranged from 8 to 22 in the five COI studies. RRs and PARFs of 18 related-health conditions compiled by Anis et al. were the most comprehensive estimates among those accessible detailed analyses [11].

### Discussion

We reviewed studies that evaluated the costs of overweight and obesity and costs of prevention programs to inform the design of economic evaluations of obesity interventions in Canada. The findings indicate that the economic burden of obesity is substantial and requires swift and comprehensive public health action. The fraction of total health care costs attributable to overweight and obesity in Canada was estimated to be as high as 12%. By contrast, there is scarce data on the costs of obesity prevention interventions in Canada to inform economic evaluations and to aid resource allocation decisions. The included cost analysis of a comprehensive school health program in Nova Scotia showed that this intervention was not resource-intensive compared to the costs of programs in other countries: For example, Carter et al. estimated the costs of various school-based obesity prevention programs in Australia to be in the range of AUS$ 28 to AUS$ 473 per student. It is important to stress that the cost of obesity treatment is considerably higher than the prevention costs. The former was estimated to be between AUS $ 650 to AUS $ 31,553 depending on the type of therapy [25]. A recent review by John et al. found heterogeneity in cost-effectiveness analyses and study quality of obesity interventions, which hampers comparison of data from different settings [10,12]. Therefore, costing should be integrated at the implementation of prevention projects and the resulting data should be made accessible for cost-effectiveness analyses.

We assessed the quality of cost analyses using the QHES and found a lack of uncertainty handling and incremental analyses as the main shortcomings of the reviewed studies. In addition, most studies did not clearly present the unit costs of obesity-related chronic conditions that limits the comparison across settings.The approach used in estimating the economic burden of obesity based on the population-attributable risks of co-morbidities is similar to previous works but the method has some drawbacks. The included COI analyses assumed that co-morbidities were mutually exclusive, and their relative risks were estimated mostly from data outside Canada.

This review identified several implications for future research. First, more bottom-up COI analyses and program costs analyses in Canadian settings are needed to guide economic evaluation of and resource allocation for obesity prevention programs. Costs analyses should include more detailed stratifications, (e.g., by sex and age), and uncertainty analysis should be used. Second, a systematic synthesis and estimate of parameters, for instance, likelihood of developing obesity overtime or unit costs of co-morbidities, using national surveys data are essential to improve the comparability and generalizability of future COI or economic evaluation studies. Finally, modeling the incidence-based lifetime costs and outcomes including direct and indirect costs from a societal perspective are essential to perform economic evaluation studies of obesity prevention programs.

We found heterogeneity in the scopes of cost analyses, including types of costs, numbers and types of related comorbidities, and BMI thresholds used. These inconsistences made it difficult to compare studies in different settings or to evaluate changes in economic burden of overweight and obesity over time. In addition, several types of costs were not determined, such as the out-of-pocket payment of households and individuals, or the costs of absenteeism to employers and employees. The varying scope of these cost analyses reflects the availability and accessibility of data sources at national and provincial levels. At the national level, Canadian Community Health Survey, National Population Health Survey and Economic Burden of Illness in Canada have good information to estimate the prevalence of obesity and its associated health care use and costs. To weigh future savings in health care costs against the costs for an obesity prevention program, an incidence-based COI analysis is required to estimate lifetime costs associated with weight status. A bottom-up costing approach and simulations using decision-analytical models are also necessary. None of the reviewed papers used longitudinal national data to estimate the changes in obesity or attempted to project total lifetime costs of obesity. This is partly due to the lack of longitudinal Canadian data on the development of weight-related health conditions and costs [26]. Nonetheless, the national data sources listed above might be used to project the changes in obesity epidemic at a population level. Further efforts to fully capture longitudinal changes in BMI trajectories and life-time costs are needed prior to economic evaluations of interventions for the prevention of obesity.

This review showed that indirect costs of obesity were substantial and account for about 45 to 60% of the total costs. Therefore, focusing solely on direct medical costs of obesity-related comorbidities does not fully capture the economic burden of the obesity epidemic. Estimating costs and monetary benefits from a societal perspective in an economic evaluation may provide a more complete picture.

Our review found only one cost analysis of a comprehensive school health program in Canada. The cost analysis was a 1-year assessment using a top-down approach. The paucity of data highlights the urgent need for cost analyses of existing and new prevention programs. When conducting an economic evaluation of an obesity prevention, considering only the savings due to reductions in excess weight does not provide a complete picture of the effect of the program. Some interventions may not change the weight status of individuals, but may still improve health status and ability to work. Second, reductions of health services utilization and health care costs as a result of an intervention might also be substantial. Consequently, in the design of an economic evaluation, the changes in costs and outcomes under intervention would also provide important additional information.

## Conclusions

To conclude, we observed high costs associated with overweight and obesity and modest costs for obesity prevention programs; however, no cost-effectiveness study of obesity interventions has been performed in Canada. Cost-effectiveness analyses of preventive programs that constitute incidence-based life-time modeling of costs and health outcomes from societal perspective are urgently needed.

## Competing interests

The authors declare that they have no competing interests.

## Authors’ contributions

PV, SK, and AO initiated the research question. BXT and AVN did the literature search and synthesis. BXT and AO appraised the quality of studies. BXT, SK, AVN, AO, and PV wrote the manuscript. All authors read and approved the final manuscript.
